# Complete Genome Sequences of Cluster A6 and Cluster G1 Mycobacterium smegmatis Phages Hoot and Jolene

**DOI:** 10.1128/MRA.00578-21

**Published:** 2021-10-21

**Authors:** Jon Thompson, Asli Özdemir, Arsen M. Topchyan, Maxwell Torosian, Victoria Z. Thymianos, Angelica Eagle, Juliana McCormick, Leon Kyle G. Boyles, Azucena A. Benito, Kurt Regner, Christy Strong, Philippos K. Tsourkas

**Affiliations:** a School of Life Sciences, University of Nevada, Las Vegas, Nevada, USA; DOE Joint Genome Institute

## Abstract

We present the complete genome sequences of Mycobacterium smegmatis phages Hoot and Jolene, isolated in Las Vegas, NV. The phages were isolated and annotated by students enrolled in an undergraduate research course at the University of Nevada, Las Vegas. Hoot is a cluster A6 mycobacteriophage, while Jolene is in cluster G1.

## ANNOUNCEMENT

Mycobacterium smegmatis is a nonpathogenic acid-fast bacterium that serves as a phage isolation host for the Science Educational Alliance Phage Hunters Advancing Genomics and Evolutionary Science (SEA-PHAGES) program (1). Here, we present the complete genome sequences of two novel M. smegmatis phages isolated by students enrolled in the SEA-PHAGES-affiliated Phage Discovery course at the University of Nevada, Las Vegas (UNLV). Seven M. smegmatis phages have been published from the three previous offerings of the course (2–4), as have four phages infecting Paenibacillus larvae that were isolated outside the course (5).

The phages were isolated from potted soil (Hoot) and Miracle Grow Potting Mix (Jolene). Environmental samples were incubated with 7H9 broth neat with CaCl_2_ and shaken (250 rpm, 2 h) at room temperature, followed by centrifugation and filter sterilization (0.22 μm) of the supernatant, as specified in the HHMI SEA-PHAGES Phage Discovery Guide (https://seaphages.org/faculty/information/#phagediscovery). The phages were purified and amplified in M. smegmatis mc^2^155. M. smegmatis mc^2^155 was grown in Middlebrook 7H9 liquid and agar plates at 37°C as described in the Phage Discovery Guide.

Phage DNA was extracted as described in the manufacturer protocol of the Norgen phage DNA isolation kit (catalog number 46800, Norgen Biotek). The phage DNA was sequenced at the University of Pittsburgh. Sequencing libraries were prepared from genomic DNA using the NEB Ultra II kit. The libraries were sequenced using the Illumina MiSeq platform, producing 150-bp single-end reads. The reads were quality trimmed and assembled *de novo* using Newbler v.2.9 with default settings, generating a single contig, which was checked for completeness, accuracy, and phage genomic termini using Consed v. 29 as described in reference 6.

The assembly results and accession numbers are listed in Table 1. The phages were assigned to a genomic cluster based on the nucleotide sequence similarity to phages in the phagesdb.org database using Clustal Omega with default settings (7, 8); Hoot is in cluster A6, while Jolene is in cluster G1. Multiple alignment of the phages in each cluster using Clustal Omega showed that both Hoot and Jolene have >99% sequence identity with multiple phages in their cluster. Both phages use the “cohesive ends with 3′ overhangs” DNA packaging strategy (9).

**TABLE 1:** Phage accession numbers and genome assembly results

Phage name	GenBank accession no.	SRA accession no.	Avg coverage (×)	Cluster	Genome length (bp)	GC content (%)	No. of genes	No. of tRNAs
Hoot	MZ171376	SRX11067173	156	A6	50,006	61.5	98	3
Jolene	MZ171377	SRX11067174	273	G1	42,305	66.7	63	0

The genes and gene starts were identified using Phage Commander v.1.0 (10) and DNA Master v. 5.23.6 (11), using the method described in reference 12. We identified 63 genes in Jolene and 98 genes in Hoot, of which 3 are tRNAs. Putative protein functions were assigned using protein BLAST (https://blast.ncbi.nlm.nih.gov/Blast.cgi?PAGE=Proteins), CD-Search (https://www.ncbi.nlm.nih.gov/Structure/bwrpsb/bwrpsb.cgi), and HHpred (https://toolkit.tuebingen.mpg.de/tools/hhpred) with default settings. Using a cutoff E value of 1E-10 for BLAST and a cutoff of 1E-4 for CD-Search and HHpred, we were able to assign putative functions to 28 genes in Jolene (44%) and 38 non-tRNA genes in Hoot (40%). A lysin A was identified in both phages; a lysin B, integrase, and excisionase were identified in Jolene but not in Hoot. The lack of an identifiable lysin B in Hoot is unusual, as most M. smegmatis phages lyse their host using a lysin A/lysin B cassette. Jolene is temperate, but despite the presence of an immunity repressor in Hoot, we did not identify a candidate for an integrase gene, raising the possibility that this phage may be virulent. The genome organization of Hoot is somewhat atypical, as there are five genes that precede the small terminase, which is followed by four genes of unknown function, in turn followed by the lysin A and holin, and then the large terminase and the structural genes.

### Data availability.

The GenBank and SRA accession numbers are listed in Table 1.
